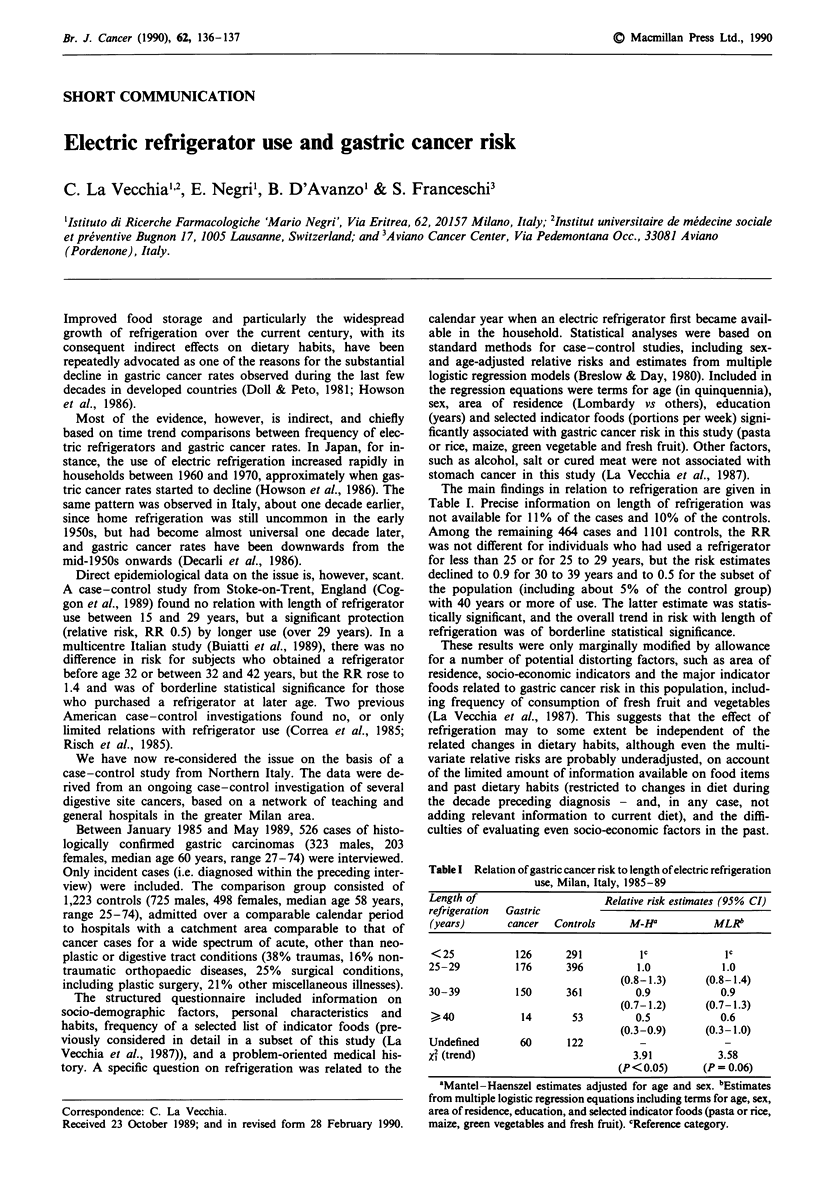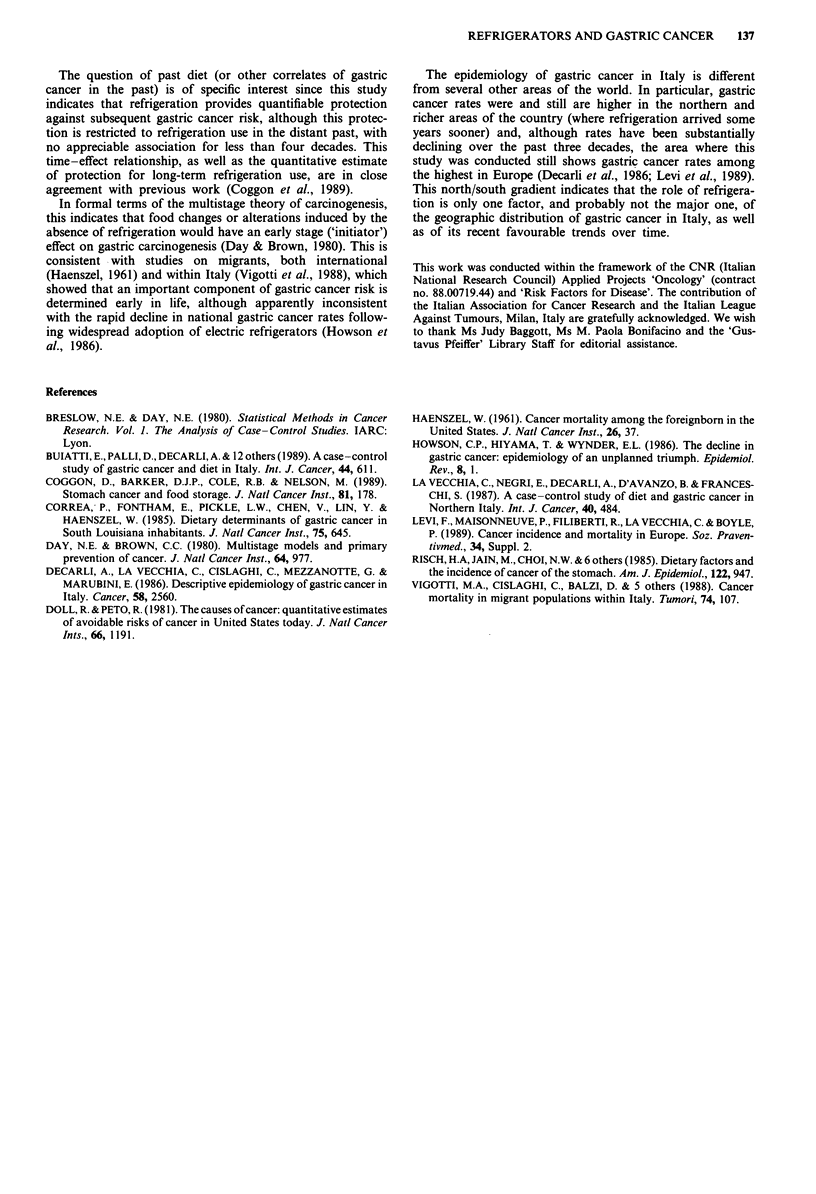# Electric refrigerator use and gastric cancer risk.

**DOI:** 10.1038/bjc.1990.245

**Published:** 1990-07

**Authors:** C. La Vecchia, E. Negri, B. D'Avanzo, S. Franceschi

**Affiliations:** Istituto di Ricerche Farmacologiche Mario Negri, Milano, Italy.


Br. J. Cancer (1990), 62, 136–137 © Macmillan Press Ltd., 1990

SHORT COMMUNICATION

### Electric refrigerator use and gastric cancer risk

C. La Vecchia[1,2], E. Negri[1], B. D'Avanzo[1] & S. Franceschi[3]

[1]*Istituto di Ricerche Farmacologiche 'Mario Negri', Via Eritrea, 62, 20157 Milano, Italy;* [2]*Institut universitaire de médecine sociale et préventive Bugnon 17, 1005 Lausanne, Switzerland; and* [3]*Aviano Cancer Center, Via Pedemontana Occ., 33081 Aviano (Pordenone), Italy.*

Improved food storage and particularly the widespread growth of refrigeration over the current century, with its consequent indirect effects on dietary habits, have been repeatedly advocated as one of the reasons for the substantial decline in gastric cancer rates observed during the last few decades in developed countries (Doll & Peto, 1981; Howson *et al.*, 1986).

Most of the evidence, however, is indirect, and chiefly based on time trend comparisons between frequency of electric refrigerators and gastric cancer rates. In Japan, for instance, the use of electric refrigeration increased rapidly in households between 1960 and 1970, approximately when gastric cancer rates started to decline (Howson *et al.*, 1986). The same pattern was observed in Italy, about one decade earlier, since home refrigeration was still uncommon in the early 1950s, but had become almost universal one decade later, and gastric cancer rates have been downwards from the mid-1950s onwards (Decarli *et al.*, 1986).

Direct epidemiological data on the issue is, however, scant. A case–control study from Stoke-on-Trent, England (Coggon *et al.*, 1989) found no relation with length of refrigerator use between 15 and 29 years, but a significant protection (relative risk, RR 0.5) by longer use (over 29 years). In a multicentre Italian study (Buiatti *et al.*, 1989), there was no difference in risk for subjects who obtained a refrigerator before age 32 or between 32 and 42 years, but the RR rose to 1.4 and was of borderline statistical significance for those who purchased a refrigerator at later age. Two previous American case–control investigations found no, or only limited relations with refrigerator use (Correa *et al.*, 1985; Risch *et al.*, 1985).

We have now re-considered the issue on the basis of a case–control study from Northern Italy. The data were derived from an ongoing case–control investigation of several digestive site cancers, based on a network of teaching and general hospitals in the greater Milan area.

Between January 1985 and May 1989, 526 cases of histologically confirmed gastric carcinomas (323 males, 203 females, median age 60 years, range 27–74) were interviewed. Only incident cases (i.e. diagnosed within the preceding interview) were included. The comparison group consisted of 1,223 controls (725 males, 498 females, median age 58 years, range 25–74), admitted over a comparable calendar period to hospitals with a catchment area comparable to that of cancer cases for a wide spectrum of acute, other than neoplastic or digestive tract conditions (38% traumas, 16% non-traumatic orthopaedic diseases, 25% surgical conditions, including plastic surgery, 21% other miscellaneous illnesses).

The structured questionnaire included information on socio-demographic factors, personal characteristics and habits, frequency of a selected list of indicator foods (previously considered in detail in a subset of this study (La Vecchia *et al.*, 1987)), and a problem-oriented medical history. A specific question on refrigeration was related to the calendar year when an electric refrigerator first became available in the household. Statistical analyses were based on standard methods for case–control studies, including sex- and age-adjusted relative risks and estimates from multiple logistic regression models (Breslow & Day, 1980). Included in the regression equations were terms for age (in quinquennia), sex, area of residence (Lombardy *vs* others), education (years) and selected indicator foods (portions per week) significantly associated with gastric cancer risk in this study (pasta or rice, maize, green vegetable and fresh fruit). Other factors, such as alcohol, salt or cured meat were not associated with stomach cancer in this study (La Vecchia *et al.*, 1987).

The main findings in relation to refrigeration are given in Table I. Precise information on length of refrigeration was not available for 11% of the cases and 10% of the controls. Among the remaining 464 cases and 1101 controls, the RR was not different for individuals who had used a refrigerator for less than 25 or for 25 to 29 years, but the risk estimates declined to 0.9 for 30 to 39 years and to 0.5 for the subset of the population (including about 5% of the control group) with 40 years or more of use. The latter estimate was statistically significant, and the overall trend in risk with length of refrigeration was of borderline statistical significance.

These results were only marginally modified by allowance for a number of potential distorting factors, such as area of residence, socio-economic indicators and the major indicator foods related to gastric cancer risk in this population, including frequency of consumption of fresh fruit and vegetables (La Vecchia *et al.*, 1987). This suggests that the effect of refrigeration may to some extent be independent of the related changes in dietary habits, although even the multivariate relative risks are probably underadjusted, on account of the limited amount of information available on food items and past dietary habits (restricted to changes in diet during the decade preceding diagnosis – and, in any case, not adding relevant information to current diet), and the difficulties of evaluating even socio-economic factors in the past.

**Table I** Relation of gastric cancer risk to length of electric refrigeration use, Milan, Italy, 1985–89

| *Length of refrigeration (years)* | *Gastric cancer* | *Controls* | *Relative risk estimates (95% CI)* M-H[a] | MLR[b] |
|---|---|---|---|---|
| <25 | 126 | 291 | 1[c] | 1[c] |
| 25–29 | 176 | 396 | 1.0 (0.8–1.3) | 1.0 (0.8–1.4) |
| 30–39 | 150 | 361 | 0.9 (0.7–1.2) | 0.9 (0.7–1.3) |
| ≥40 | 14 | 53 | 0.5 (0.3–0.9) | 0.6 (0.3–1.0) |
| Undefined | 60 | 122 | – | – |
| $\chi_1^2$ (trend) | | | 3.91 ($P<0.05$) | 3.58 ($P=0.06$) |

[a]Mantel–Haenszel estimates adjusted for age and sex. [b]Estimates from multiple logistic regression equations including terms for age, sex, area of residence, education, and selected indicator foods (pasta or rice, maize, green vegetables and fresh fruit). [c]Reference category.

Correspondence: C. La Vecchia.
Received 23 October 1989; and in revised form 28 February 1990.

The question of past diet (or other correlates of gastric cancer in the past) is of specific interest since this study indicates that refrigeration provides quantifiable protection against subsequent gastric cancer risk, although this protection is restricted to refrigeration use in the distant past, with no appreciable association for less than four decades. This time–effect relationship, as well as the quantitative estimate of protection for long-term refrigeration use, are in close agreement with previous work (Coggon *et al.*, 1989).

In formal terms of the multistage theory of carcinogenesis, this indicates that food changes or alterations induced by the absence of refrigeration would have an early stage ('initiator') effect on gastric carcinogenesis (Day & Brown, 1980). This is consistent with studies on migrants, both international (Haenszel, 1961) and within Italy (Vigotti *et al.*, 1988), which showed that an important component of gastric cancer risk is determined early in life, although apparently inconsistent with the rapid decline in national gastric cancer rates following widespread adoption of electric refrigerators (Howson *et al.*, 1986).

The epidemiology of gastric cancer in Italy is different from several other areas of the world. In particular, gastric cancer rates were and still are higher in the northern and richer areas of the country (where refrigeration arrived some years sooner) and, although rates have been substantially declining over the past three decades, the area where this study was conducted still shows gastric cancer rates among the highest in Europe (Decarli *et al.*, 1986; Levi *et al.*, 1989). This north/south gradient indicates that the role of refrigeration is only one factor, and probably not the major one, of the geographic distribution of gastric cancer in Italy, as well as of its recent favourable trends over time.

This work was conducted within the framework of the CNR (Italian National Research Council) Applied Projects 'Oncology' (contract no. 88.00719.44) and 'Risk Factors for Disease'. The contribution of the Italian Association for Cancer Research and the Italian League Against Tumours, Milan, Italy are gratefully acknowledged. We wish to thank Ms Judy Baggott, Ms M. Paola Bonifacino and the 'Gustavus Pfeiffer' Library Staff for editorial assistance.

### References

BRESLOW, N.E. & DAY, N.E. (1980). *Statistical Methods in Cancer Research. Vol. 1. The Analysis of Case–Control Studies.* IARC: Lyon.

BUIATTI, E., PALLI, D., DECARLI, A. & 12 others (1989). A case–control study of gastric cancer and diet in Italy. *Int. J. Cancer*, **44**, 611.

COGGON, D., BARKER, D.J.P., COLE, R.B. & NELSON, M. (1989). Stomach cancer and food storage. *J. Natl Cancer Inst.*, **81**, 178.

CORREA, P., FONTHAM, E., PICKLE, L.W., CHEN, V., LIN, Y. & HAENSZEL, W. (1985). Dietary determinants of gastric cancer in South Louisiana inhabitants. *J. Natl Cancer Inst.*, **75**, 645.

DAY, N.E. & BROWN, C.C. (1980). Multistage models and primary prevention of cancer. *J. Natl Cancer Inst.*, **64**, 977.

DECARLI, A., LA VECCHIA, C., CISLAGHI, C., MEZZANOTTE, G. & MARUBINI, E. (1986). Descriptive epidemiology of gastric cancer in Italy. *Cancer*, **58**, 2560.

DOLL, R. & PETO, R. (1981). The causes of cancer: quantitative estimates of avoidable risks of cancer in United States today. *J. Natl Cancer Ints.*, **66**, 1191.

HAENSZEL, W. (1961). Cancer mortality among the foreignborn in the United States. *J. Natl Cancer Inst.*, **26**, 37.

HOWSON, C.P., HIYAMA, T. & WYNDER, E.L. (1986). The decline in gastric cancer: epidemiology of an unplanned triumph. *Epidemiol. Rev.*, **8**, 1.

LA VECCHIA, C., NEGRI, E., DECARLI, A., D'AVANZO, B. & FRANCESCHI, S. (1987). A case–control study of diet and gastric cancer in Northern Italy. *Int. J. Cancer*, **40**, 484.

LEVI, F., MAISONNEUVE, P., FILIBERTI, R., LA VECCHIA, C. & BOYLE, P. (1989). Cancer incidence and mortality in Europe. *Soz. Praventivmed.*, **34**, Suppl. 2.

RISCH, H.A, JAIN, M., CHOI, N.W. & 6 others (1985). Dietary factors and the incidence of cancer of the stomach. *Am. J. Epidemiol.*, **122**, 947.

VIGOTTI, M.A., CISLAGHI, C., BALZI, D. & 5 others (1988). Cancer mortality in migrant populations within Italy. *Tumori*, **74**, 107.